# Supplementary material for: COLOFIT: Development and Internal‐External Validation of Models Using Age, Sex, Faecal Immunochemical and Blood Tests to Optimise Diagnosis of Colorectal Cancer in Symptomatic Patients
Source: Aliment Pharmacol Ther. 2025 Jan 7;61(5):852–64. doi: 10.1111/apt.18459 (PMC11825929; doi:10.1111/apt.18459)
Supplement: Supplementary file 1 — Data S1. [file APT-61-852-s001.docx]

# Supplementary

[Supplementary 1](#_Toc150939446)

[Supplementary results 3](#_Toc150939447)

[Logistic Regression model 3](#_Toc150939448)

[Predictor Model building 3](#_Toc150939449)

[*Logistic Model* 3](#_Toc150939450)

[Performance in the validation cohort 3](#_Toc150939451)

[Logistic Model 3](#_Toc150939452)

[Stratified C statistic performance 3](#_Toc150939453)

[Calibration 3](#_Toc150939454)

[Performance 3](#_Toc150939455)

[Net benefit analysis 3](#_Toc150939456)

[Figure S1. Mixing in the derivation cohort for multiple imputation using chained equations with predictive mean matching, with a random intercept for each patient and time from test fitted as a within patient gradient 4](#_Toc150939457)

[Figure S2. Mixing in the validation cohort for multiple imputation using chained equations using chained equations with predictive mean matching, with a random intercept for each patient and time from test fitted as a within patient gradient 4](#_Toc150939458)

[Figure S3. Fractional polynomial transformation of age compared to crude risk of colorectal cancer by age category 5](#_Toc150939459)

[Figure S4. Fractional polynomial transformation of FIT compared to crude risk of colorectal cancer by FIT category 6](#_Toc150939460)

[Figure S5. Fractional polynomial transformation of platelet count compared to crude risk of colorectal cancer by platelet count category 6](#_Toc150939461)

[Table S1. Fitted Cox proportional hazards survival model. Transformations selected for Cox Survival model using backwards multiple fractional polynomial algorithm with weighted likelihood ratio testing across the stacked imputed datasets, keeping the familywise error rate at p = 0.05 7](#_Toc150939462)

[Table S2. Survival model for one year survival probability from colorectal cancer 7](#_Toc150939463)

[Table S3: Generalised likelihood ratio testing of interactions between the transformed variables in the Cox survival model in the derivation cohort (p-values) 7](#_Toc150939464)

[Table S4. Fitted logistic regression model. Transformations selected for logistic model using backwards multiple fractional polynomial algorithm with weighted likelihood ratio testing across the stacked imputed datasets, keeping the familywise error rate at p = 0.05 7](#_Toc150939465)

[Table S5. Logistic model for one-year survival probability from colorectal cancer 8](#_Toc150939466)

[Figure S6. Calibration plots. Blue line is linear fit to these estimates with intercept and gradient shown. 8](#_Toc150939467)

[Figure S7. Calibration plots. Blue line is linear fit to these estimates with intercept and gradient shown. 9](#_Toc150939468)

[Table S6 Fitted Cox proportional hazards survival model without including blood tests. Transformations selected for Cox Survival model using backwards multiple fractional polynomial algorithm with weighted likelihood ratio testing across the stacked imputed datasets, keeping the familywise error rate at p = 0.05 9](#_Toc150939469)

[Table S7 Fitted logistic regression model without including blood tests. Transformations selected for logistic model using backwards multiple fractional polynomial algorithm with weighted likelihood ratio testing across the stacked imputed datasets, keeping the familywise error rate at p = 0.05 9](#_Toc150939470)

[Table S8. Stratified C statistics calculated for the logistic model and pooled across 10 imputed datasets in the derivation and validation cohorts 9](#_Toc150939471)

[Table S9. Positive predictive value, negative predictive value, sensitivity, and specificity, at different thresholds for predicted one-year colorectal cancer risk. Logistic regression model using multiple fractional polynomial transformations compared to a binary FIT cut off at 10 and 40. 10](#_Toc150939472)

[Derivation. FIT tests 1st Nov 2016- 30th Nov 2021 Validation: FIT tests 1st Dec 2021 –Nov 2022 11](#_Toc150939473)

[Table S9 Extrapolating true and false positive and negative rates from logistic model to 100,000 FIT tests in the validation cohort 11](#_Toc150939474)

Supplementary results

Logistic Regression model

### Predictor Model building

*Logistic Model*

In the logistic model the same multiple fractional polynomial transformations were selected for age, FIT, and platelet count as in the survival model with MCV included as a linear variable and sex as a binary variable. The fitted model is shown in table S4, with the fitted equation predicting one-year survival from CRC in table S5. The area under the receiver operator curve for the model in the derivation cohort was C = 0.934 (0.929-0.939).

### Performance in the validation cohort

#### Logistic Model

##### Stratified C statistic performance

Concordance across the derivation and validation cohorts remained highest in younger patients, again with no clear drop in performance in the validation cohort (Table S8). Similarly, the performance did not drop across the ethnicities accepting reduced power for Black and Other ethnicity strata from fewer events.

##### Calibration

Figure S7 shows there was a reduction in the calibration of the logistic model similar to the survival model in the validation cohort compared to the derivation, but it remained acceptable and did not need recalibrating allowing for the increased variability from smaller numbers.

##### Performance

Table S9 shows the positive predictive value, negative predictive value, sensitivity and specificity of the logistic model compared to a FIT 10 and FIT 40 cut off in the derivation and validation cohort. The logistic model with blood tests had a similar 1% to 2% increase in the positive predictive value compared to a binary FIT cut off or a logistic model with FIT, age and sex model (supplementary table S7) compared to the Cox model with blood tests, with a similar negative predictive value. This was reflected in the improvement in specificity at the expense of a reduction in sensitivity using the model compared to binary FIT cut offs. However, the overall positive rate from the logistic model was similar to the Cox model with blood tests, with a slightly lower sensitivity.

##### Net benefit analysis

Figure S8 shows the net benefit plots for the logistic regression model which compares the balance between true positives and false positives weighted for the different cancer threshold probabilities that can trigger referral to secondary care. Like the survival analysis this shows that at all thresholds there was a net benefit of using the model compared to FIT only. Extrapolating true and false positive and negative rates to 100,000 FIT tests in the validation cohort showed that using the model with blood tests reduced the number of negative and unnecessary colonoscopies performed by 2000-12000 (24-44% reduction) compared to just using >10 FIT cut off. However, there was an increase of 18-43 (17-20% increase) missed cancers due to the slightly lower overall positive rate and sensitivity of the logistic model with blood tests (Table S10).

Figure S1. Mixing in the derivation cohort for multiple imputation using chained equations with predictive mean matching, with a random intercept for each patient and time from test fitted as a within patient gradient
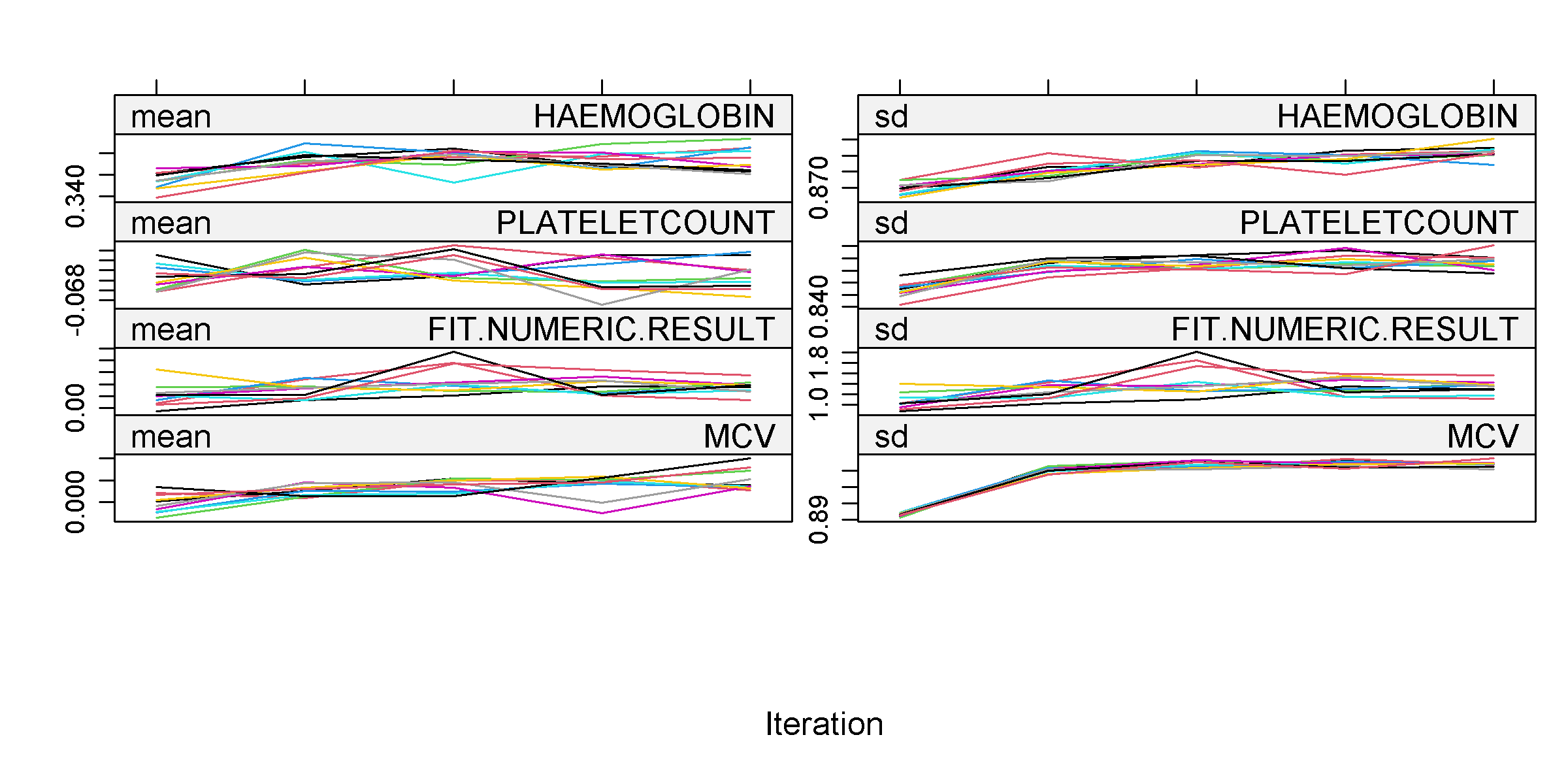


###### Figure S2. Mixing in the validation cohort for multiple imputation using chained equations using chained equations with predictive mean matching, with a random intercept for each patient and time from test fitted as a within patient gradient


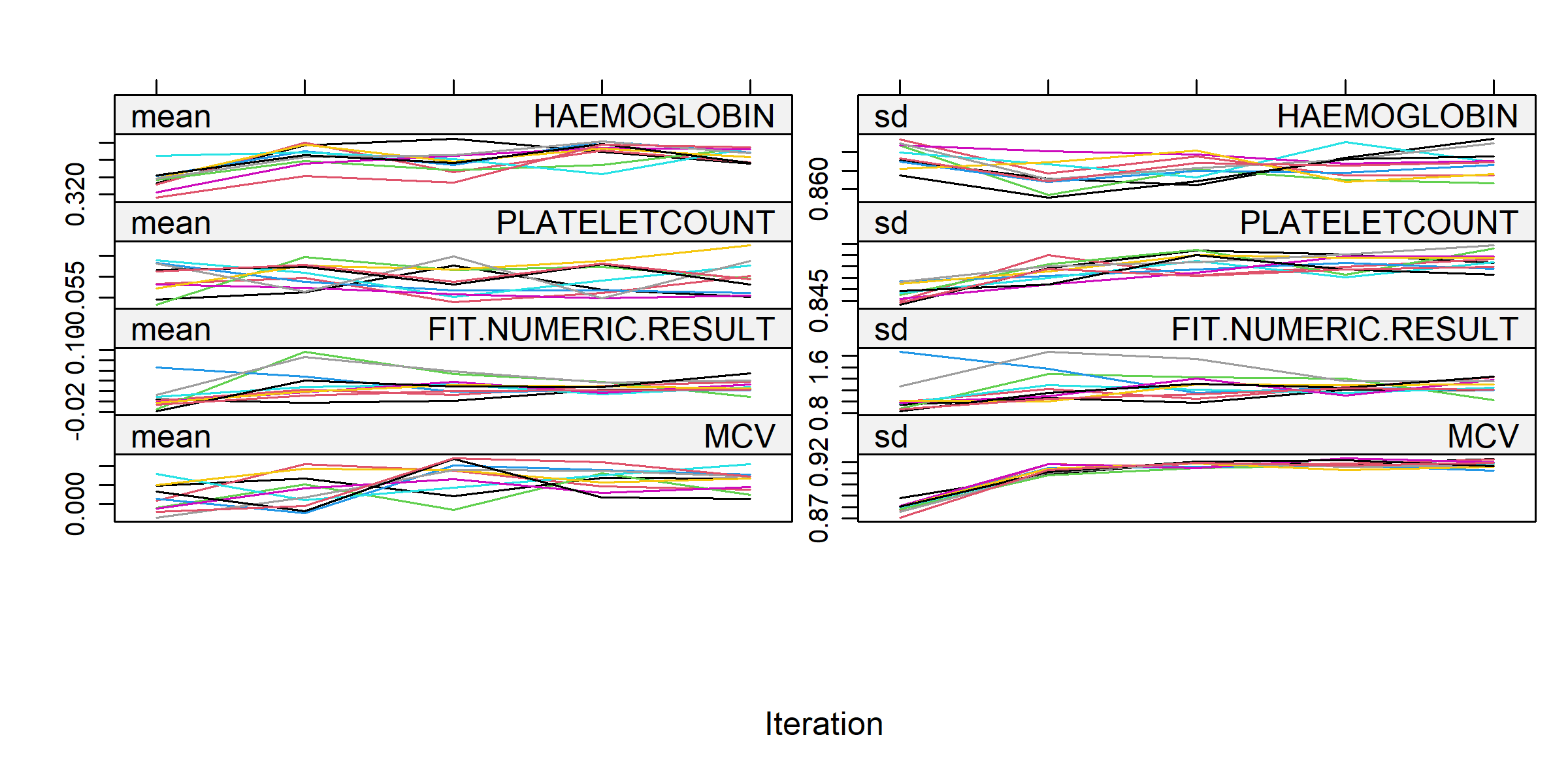


###### Figure S3. Fractional polynomial transformation of age compared to crude risk of colorectal cancer by age category


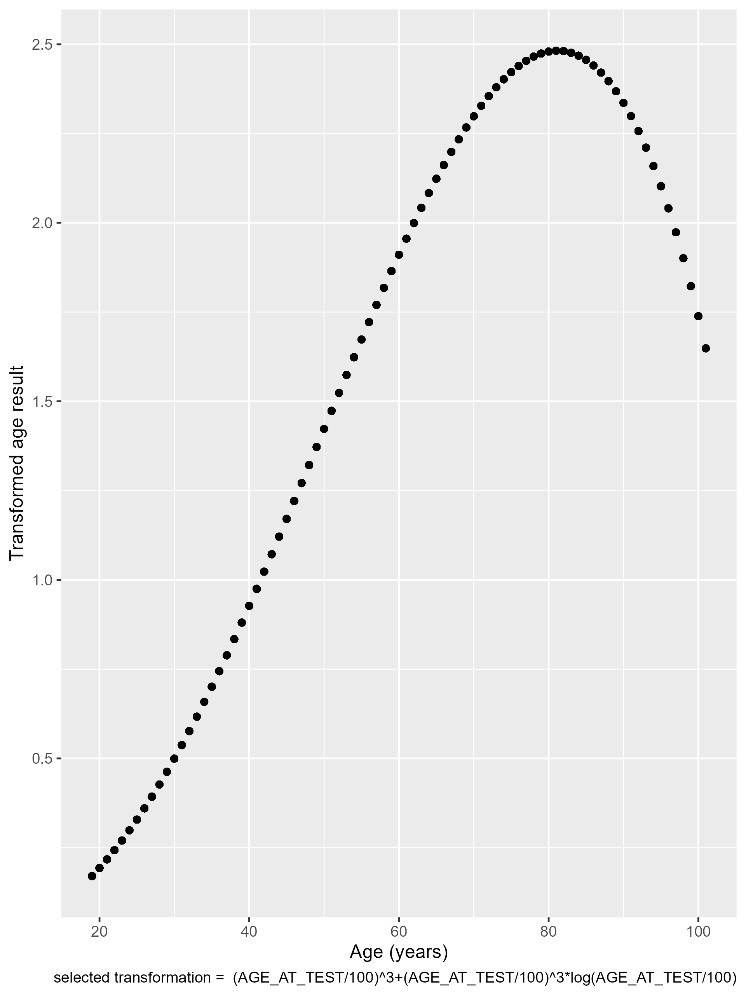

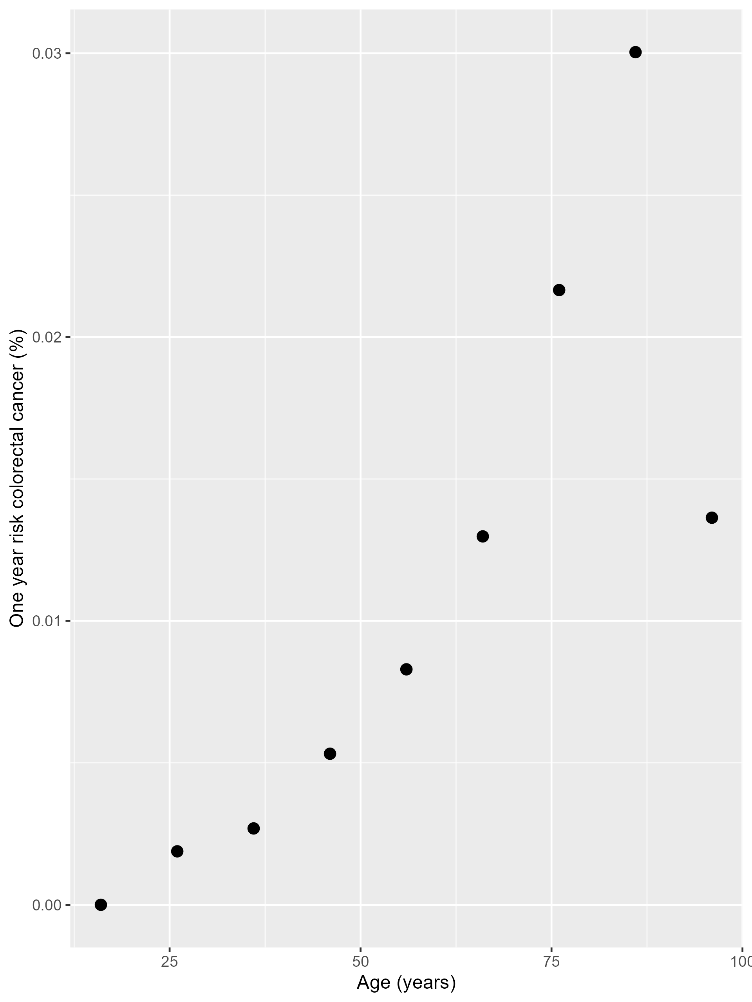


###### Figure S4. Fractional polynomial transformation of FIT compared to crude risk of colorectal cancer by FIT category


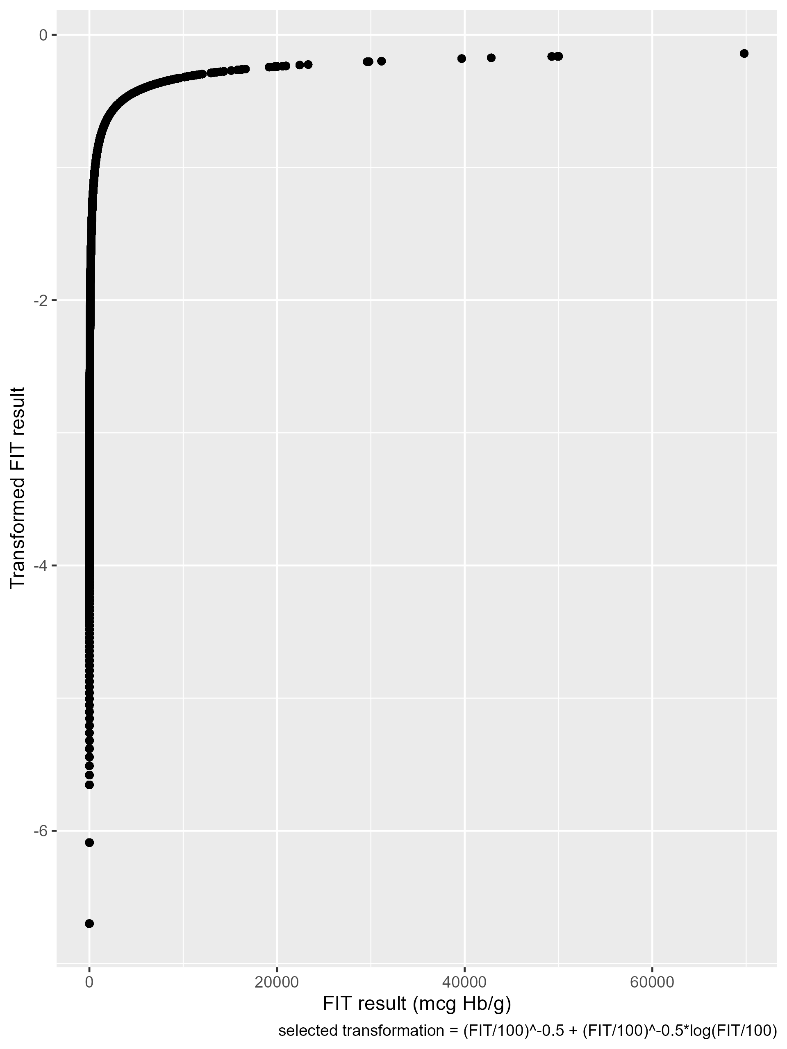

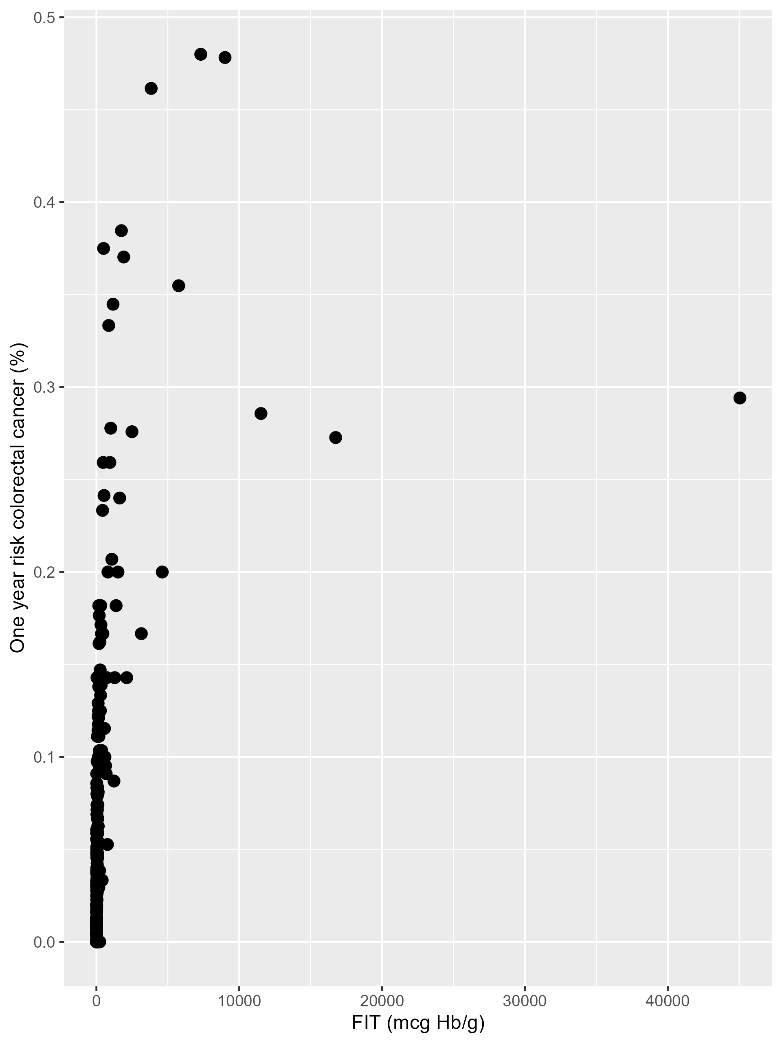


###### Figure S5. Fractional polynomial transformation of platelet count compared to crude risk of colorectal cancer by platelet count category


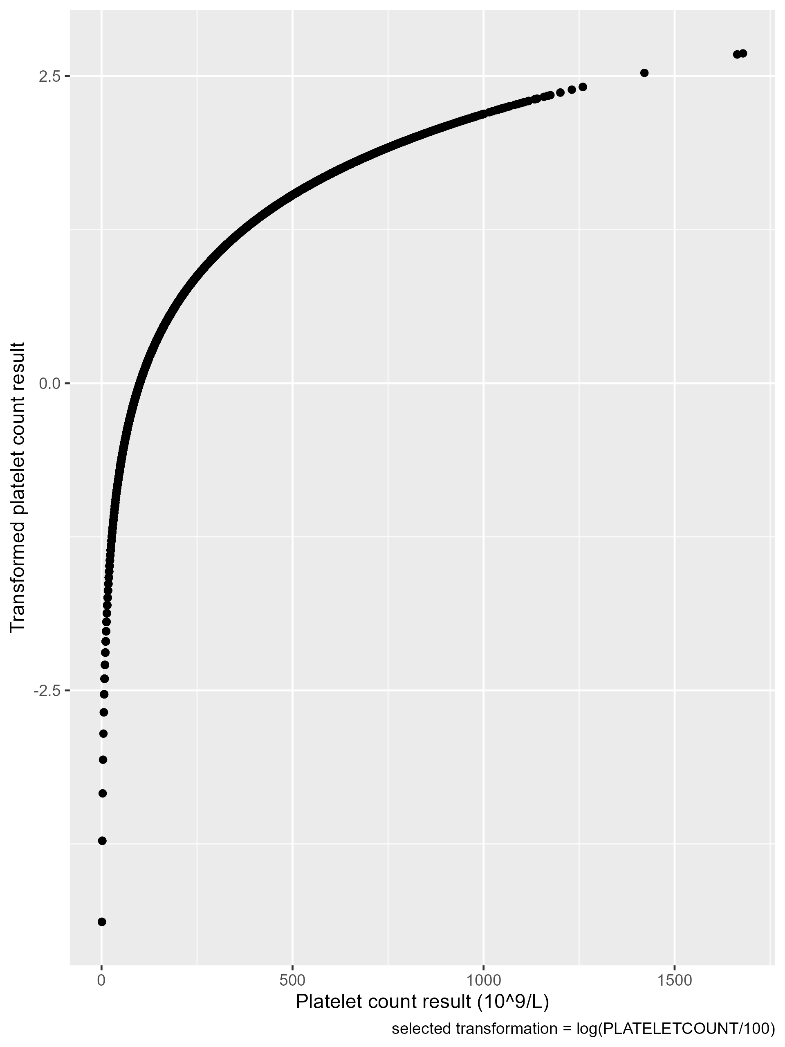

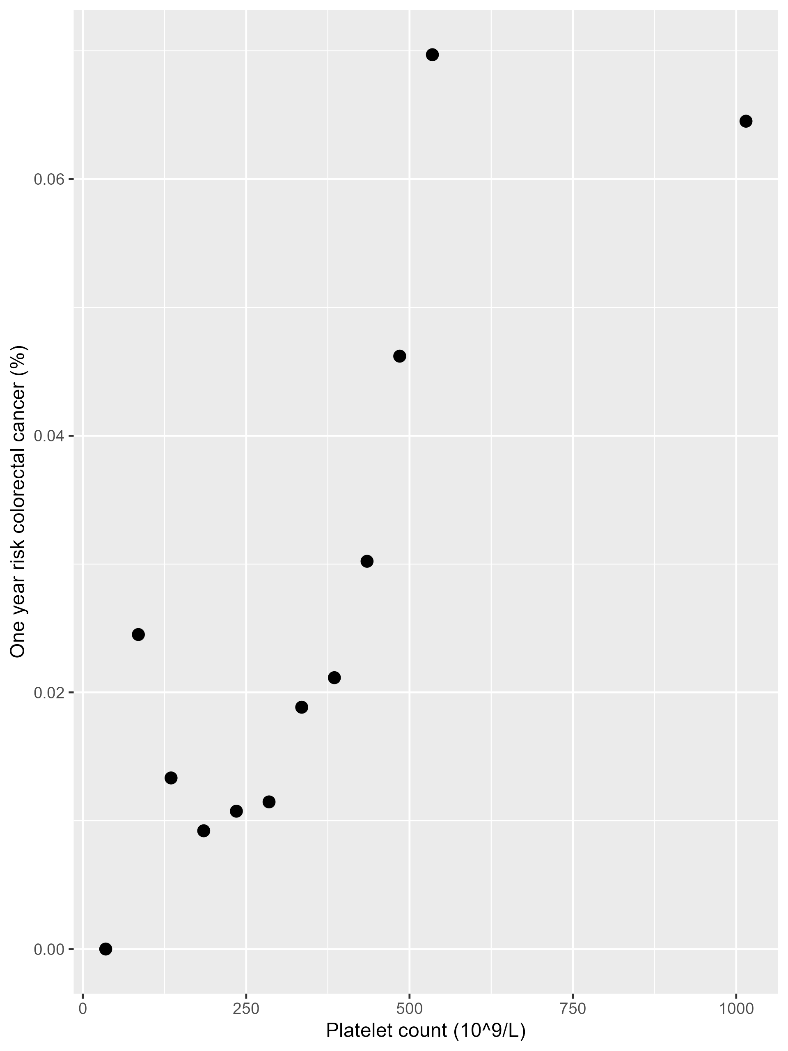


###### Table S1. Fitted Cox proportional hazards survival model. Transformations selected for Cox Survival model using backwards multiple fractional polynomial algorithm with weighted likelihood ratio testing across the stacked imputed datasets, keeping the familywise error rate at p = 0.05

| *Predictors* | *Hazard ratio* | *95% Confidence Interval* | *p-value* |
| --- | --- | --- | --- |
| (Age at test/100)^3 | 5.30 | 4.50 – 6.25 | **<0.001** |
| (Age at test/100)^3* log(Age at test/100) | 8.8e-7 | 1.6e-7 – 4.75e-6 | **<0.001** |
| (FIT/100)^-0.5 | 0.14 | 0.12 – 0.15 | **<0.001** |
| (FIT/100)^-0.5 * log(FIT/100) | 0.77 | 0.75 – 0.79 | **<0.001** |
| log(PLATELETCOUNT/100) | 2.51 | 2.32 – 2.72 | **<0.001** |
| MCV/100 | 0.02 | 0.01 – 0.03 | **<0.001** |
| Gender (Male) | 1.58 | 1.49 – 1.67 | **<0.001** |

###### Table S2. Survival model for one year survival probability from colorectal cancer

$$\left( e^{-0.6592014} \right)^{\left( \begin{aligned} + 1.6685765 \left( \frac{AGE}{100} \right)^{3}-13.9435406\left( \frac{AGE}{100} \right)^{3}\cdot\ln\left( \frac{AGE}{100} \right) \\ \\ -\frac{1.9965475}{\sqrt{\left( \frac{FIT}{100} \right)}} -\frac{0.2657153}{\sqrt{\left( \frac{FIT}{100} \right)}} \cdot\ln\left( \frac{FIT}{100} \right) \\ \\ +0.9208493\cdot ln \begin{aligned} \left( \frac{PLATELETS}{100} \right) \end{aligned} \\ \\ -\frac{3.9007829 \cdot MCV}{100} \\ \\ \\ +0.4543275 \cdot male \end{aligned} \right)}$$

###### Table S3: Generalised likelihood ratio testing of interactions between the transformed variables in the Cox survival model in the derivation cohort (p-values)

| *P value for LRT* | Age | FIT | Platelet | MCV |
| --- | --- | --- | --- | --- |
| Gender | 0.94 | 0.67 | 0.69 | 0.99 |
| Age |  | 0.33 | 0.67 | 0.12 |
| FIT |  |  | 1.00 | 0.97 |
| Platelet |  |  |  | 0.69 |

###### Table S4. Fitted logistic regression model. Transformations selected for logistic model using backwards multiple fractional polynomial algorithm with weighted likelihood ratio testing across the stacked imputed datasets, keeping the familywise error rate at p = 0.05

| *Predictors* | *Odds ratio* | *95% Confidence Interval* | *p-value* |
| --- | --- | --- | --- |
| (Age at test/100)^3 | 7.12 | 5.99 – 8.49 | **<0.001** |
| (Age at test/100)^3* log(Age at test/100) | 2.8e-7 | 4.6e-8 – 1.6e-6 | **<0.001** |
| (FIT/100)^-0.5 | 0.12 | 0.10 – 0.12 | **<0.001** |
| (FIT/100)^-0.5 * log(FIT/100) | 0.73 | 0.71 – 0.75 | **<0.001** |
| log(PLATELETCOUNT/100) | 2.92 | 2.69 – 3.2 | **<0.001** |
| MCV/100 | 0.009 | 0.006 – 0.013 | **<0.001** |
| Gender (Male) | 1.67 | 1.49 – 1.67 | **<0.001** |

###### Table S5. Logistic model for one-year probability of colorectal cancer

$$logistic\left( \begin{aligned} +0.1216817 \\ \\ + 1.96315 \left( \frac{AGE}{100} \right)^{3}-15.09326\left( \frac{AGE}{100} \right)^{3}\cdot\ln\left( \frac{AGE}{100} \right) \\ \\ -\frac{2.19346}{\sqrt{\left( \frac{FIT}{100} \right)}} -\frac{0.31620}{\sqrt{\left( \frac{FIT}{100} \right)}} \cdot\ln\left( \frac{FIT}{100} \right) \\ \\ +1.07231 \cdot\ln\left( \frac{PLATELETS}{100} \right) \\ \\ \\ -\frac{4.73172 \cdot MCV}{100} \\ \\ +0.51152 \cdot male \end{aligned} \right)$$

###### Figure S6. Calibration plots of Cox survival models. Blue line is linear fit to these estimates with intercept and gradient shown.

Derivation. FIT tests 1st Nov 2016- 30th Nov 2021 Validation: FIT tests 1st Dec 2021 – 30th Nov 2022


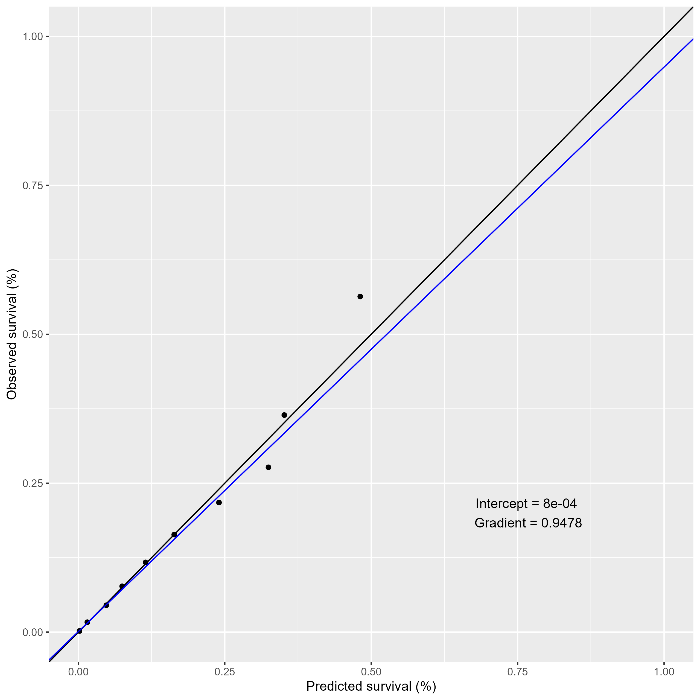

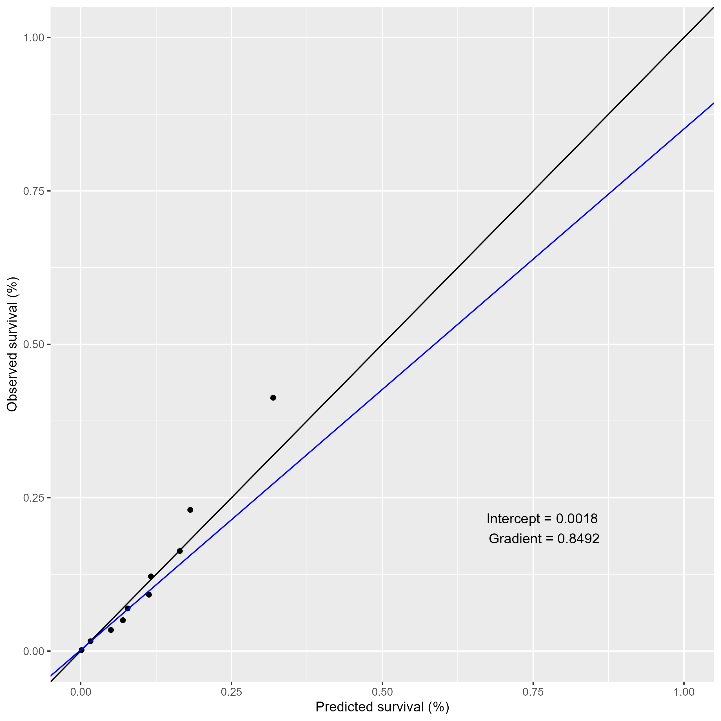


###### Figure S7. Calibration plots of logistic models. Blue line is linear fit to these estimates with intercept and gradient shown.

Derivation. FIT tests 1st Nov 2016- 30th Nov 2021 Validation: FIT tests 1st Dec 2021 –30^th^ Nov 2022


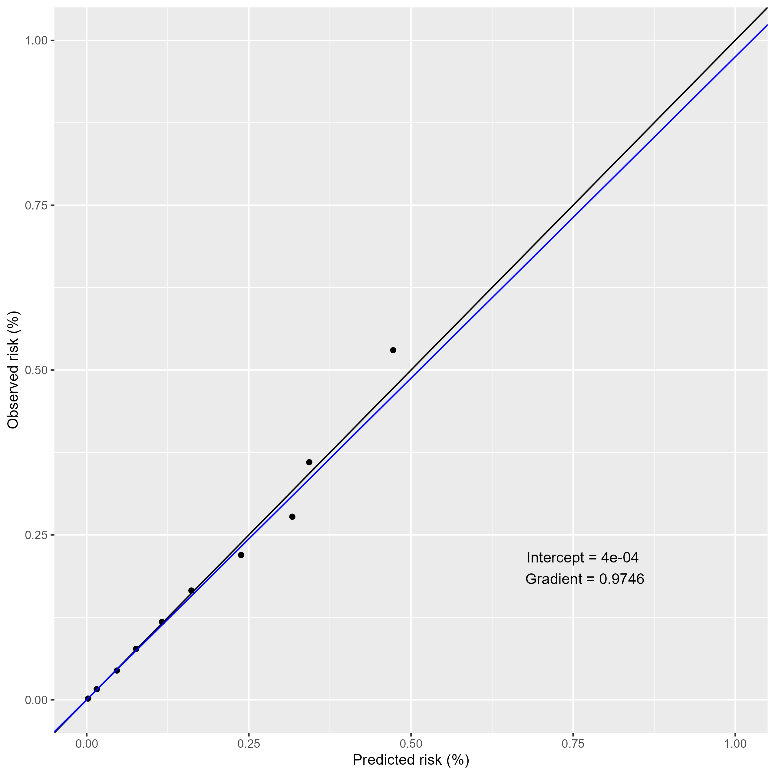

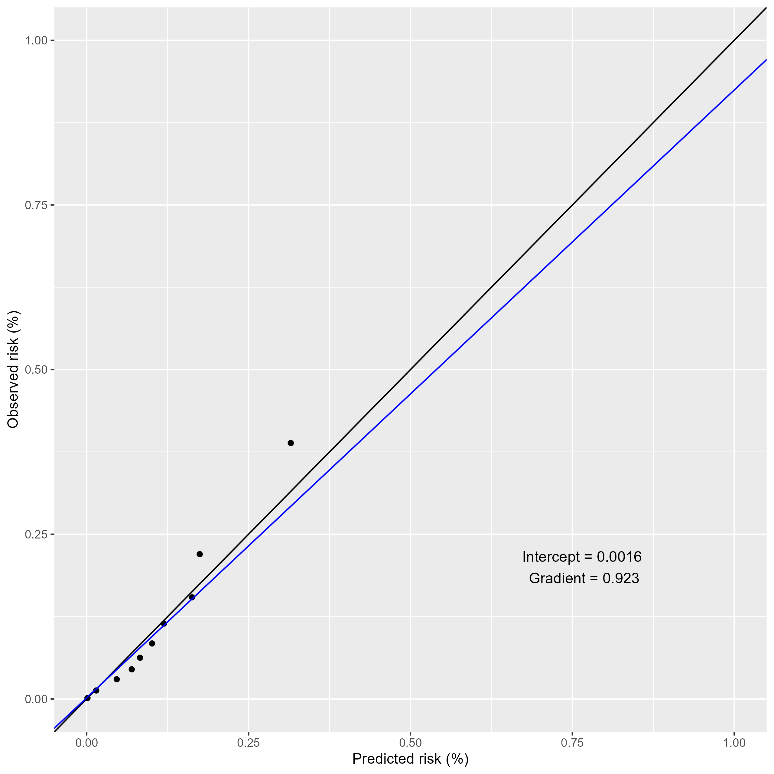


###### Table S6 Fitted Cox proportional hazards survival model without including blood tests. Transformations selected for Cox Survival model using backwards multiple fractional polynomial algorithm with weighted likelihood ratio testing across the stacked imputed datasets, keeping the familywise error rate at p = 0.05

| Predictors | Hazard ratio | conf.low | conf.high | p.value |
| --- | --- | --- | --- | --- |
| (AGE_AT_TEST/100)^3 | 4.55 | 3.87 | 5.35 | <0.001 |
| (AGE_AT_TEST/100)^3 * log((AGE_AT_TEST/100) | 5.88E-07 | 1.10E-07 | 3.13E-06 | <0.001 |
| (FIT.NUMERIC.RESULT/100)^-0.5 | 0.12 | 0.11 | 0.13 | <0.001 |
| (FIT.NUMERIC.RESULT/100)^-0.5 * log(FIT.NUMERIC.RESULT/100) | 0.74 | 0.72 | 0.76 | <0.001 |
| gender | 1.31 | 1.24 | 1.38 | <0.001 |

###### Table S7 Fitted logistic regression model without including blood tests. Transformations selected for logistic model using backwards multiple fractional polynomial algorithm with weighted likelihood ratio testing across the stacked imputed datasets, keeping the familywise error rate at p = 0.05

| Predictors | Odds ratio | conf.low | conf.high | p.value |
| --- | --- | --- | --- | --- |
| Intercept | 0.0061 | 0.0048 | 0.0077 | <0.001 |
| (AGE_AT_TEST/100)^3 | 5.39 | 4.54 | 6.42 | <0.001 |
| (AGE_AT_TEST/100)^3 * log((AGE_AT_TEST/100) | 1.97E-07 | 3.20E-08 | 1.17E-06 | <0.001 |
| log(FIT.NUMERIC.RESULT/100) | 2.31 | 2.28 | 2.35 | <0.001 |
| log(FIT.NUMERIC.RESULT/100)^2 | 0.92 | 0.91 | 0.92 | <0.001 |
| gender | 1.35 | 1.27 | 1.44 | <0.001 |

###### Table S8. Stratified C statistics calculated for the logistic model and pooled across 10 imputed datasets in the derivation and validation cohorts

|  | Derivation. FIT tests 1st November 2016- 30th November 2021 | | | Validation: FIT tests 1st December 2021 - 30th November 2022 | | | |
| --- | --- | --- | --- | --- | --- | --- | --- |
| Strata | **N** | **C statistic** | **95% CI** | **N** | **C statistic** | **95% CI** | |
| 18-50 years | 6093 | 0.96 | 0.92 to 1 | 4742 | 0.94 | 0.87 to 1.01 | |
| 51-70 years | 14066 | 0.95 | 0.93 to 0.97 | 6634 | 0.92 | 0.89 to 0.95 | |
| 71-80 years | 8348 | 0.92 | 0.89 to 0.94 | 3257 | 0.93 | 0.9 to 0.96 | |
| >80 years | 5724 | 0.89 | 0.87 to 0.91 | 2102 | 0.91 | 0.87 to 0.95 | |
| White | 24223 | 0.95 | 0.93 to 0.96 | 10903 | 0.93 | | 0.9 to 0.95 |
| Asian | 1458 | 0.93 | 0.88 to 0.99 | 783 | 0.95 | | 0.92 to 0.99 |
| Black | 853 | 0.9 | 0.79 to 1 | 476 | 0.94 | | 0.85 to 1.02 |
| Other | 658 | 0.9 | 0.78 to 1 | 405 | 0.92 | | 0.74 to 1.1 |
| Not recorded | 7039 | 0.92 | 0.88 to 0.96 | 4168 | 0.94 | | 0.91 to 0.97 |

###### Table S9. Positive predictive value, negative predictive value, sensitivity, and specificity, at different thresholds for predicted one-year colorectal cancer risk. Logistic regression model using multiple fractional polynomial transformations compared to a binary FIT cut off at 10 and 40.

| Selected cut off for referral for further investigations | | Derivation. FIT tests 1st November 2016- 30th November 2021 | | | | Validation: FIT tests 1st December 2021 - June 1st 2022 | | | |
| --- | --- | --- | --- | --- | --- | --- | --- | --- | --- |
| Cancer risk  threshold (Kaplan Meier estimate) | **Equivalent FIT only threshold approximated by linear interpolation** | **Positive Predictive Value from logistic model** | **Negative Predictive Value from logistic model** | **Sensitivity of logistic model** | **Specificity of logistic model** | **Positive Predictive Value from logistic model** | **Negative Predictive Value from logistic model** | **Sensitivity of logistic model** | **Specificity of logistic model** |
| FIT, blood tests, age and sex | |  |  |  |  |  |  |  |  |
| 0.64% | FIT=10 | 0.0647 (0.059 to 0.0703) | 0.9984 (0.9978 to 0.9988) | 0.9141 (0.8884 to 0.9379) | 0.7976 (0.7931 to 0.8022) | 0.0636 (0.0609 to 0.0664) | 0.999 (0.998 to 0.999) | 0.912 (0.899 to 0.925) | 0.833 (0.831 to 0.835) |
| 1% | FIT=13 | 0.0795 (0.0725 to 0.0866) | 0.9979 (0.9973 to 0.9984) | 0.8822 (0.8522 to 0.9104) | 0.8437 (0.8396 to 0.8478) | 0.0783 (0.075 to 0.0818) | 0.999 (0.998 to 0.999) | 0.898 (0.885 to 0.911) | 0.868 (0.867 to 0.87) |
| 2% | FIT=28 | 0.1046 (0.0953 to 0.1139) | 0.9973 (0.9966 to 0.9978) | 0.8401 (0.8063 to 0.8727) | 0.89 (0.8865 to 0.8935) | 0.102 (0.0973 to 0.106) | 0.998 (0.998 to 0.998) | 0.859 (0.844 to 0.874) | 0.905 (0.904 to 0.907) |
| 3% | **FIT=40** | 0.1252 (0.1138 to 0.1366) | 0.9968 (0.9961 to 0.9974) | 0.8072 (0.7709 to 0.8417) | 0.9137 (0.9105 to 0.9168) | 0.117 (0.112 to 0.123) | 0.997 (0.997 to 0.997) | 0.795 (0.777 to 0.812) | 0.926 (0.924 to 0.927) |
| FIT, age and sex | |  |  |  |  |  |  |  |  |
| 0.64% | FIT=10 | 0.0632 (0.0577 to 0.0687) | 0.9982 (0.9977 to 0.9987) | 0.9089 (0.8833 to 0.932) | 0.7938 (0.7894 to 0.798) | 0.0553 (0.0529 to 0.0577) | 0.999 (0.999 to 0.999) | 0.922 (0.911 to 0.934) | 0.803 (0.802 to 0.805) |
| 1% | FIT=13 | 0.0762 (0.0695 to 0.083) | 0.9977 (0.9971 to 0.9982) | 0.874 (0.8448 to 0.9018) | 0.8378 (0.8338 to 0.8417) | 0.0694 (0.0664 to 0.0723) | 0.999 (0.998 to 0.999) | 0.903 (0.889 to 0.915) | 0.849 (0.847 to 0.851) |
| 2% | FIT=28 | 0.0994 (0.0905 to 0.1085) | 0.997 (0.9963 to 0.9976) | 0.8236 (0.7908 to 0.8555) | 0.8858 (0.8824 to 0.8892) | 0.0883 (0.0844 to 0.092) | 0.998 (0.998 to 0.998) | 0.864 (0.849 to 0.878) | 0.889 (0.887 to 0.89) |
| 3% | **FIT=40** | 0.1189 (0.1081 to 0.1296) | 0.9966 (0.9959 to 0.9972) | 0.7946 (0.7592 to 0.8293) | 0.9099 (0.9068 to 0.913) | 0.101 (0.0966 to 0.105) | 0.998 (0.997 to 0.998) | 0.825 (0.808 to 0.84) | 0.909 (0.907 to 0.91) |
| FIT only model | |  |  |  |  |  |  |  |  |
| FIT ≥ 10 | | 0.0602 (0.0549 to 0.0655) | 0.998 (0.9974 to 0.9985) | 0.8953 (0.868 to 0.9207) | 0.786 (0.7817 to 0.7903) | 0.0374 (0.0358 to 0.039) | 0.999 (0.998 to 0.999) | 0.927 (0.916 to 0.938) | 0.703 (0.701 to 0.705) |
| FIT ≥ 40 | | 0.1159 (0.1053 to 0.1265) | 0.9962 (0.9955 to 0.9969) | 0.7733 (0.7366 to 0.8086) | 0.9097 (0.9066 to 0.9127) | 0.0958 (0.0916 to 0.1) | 0.998 (0.997 to 0.998) | 0.83 (0.814 to 0.846) | 0.902 (0.901 to 0.904) |

Figure S8. Net benefit plots comparing the developed logistic model against FIT only logistic models at different cancer risk (%) referral thresholds.

###### Derivation. FIT tests 1st Nov 2016- 30th Nov 2021 Validation: FIT tests 1st Dec 2021 – 30th Nov 2022


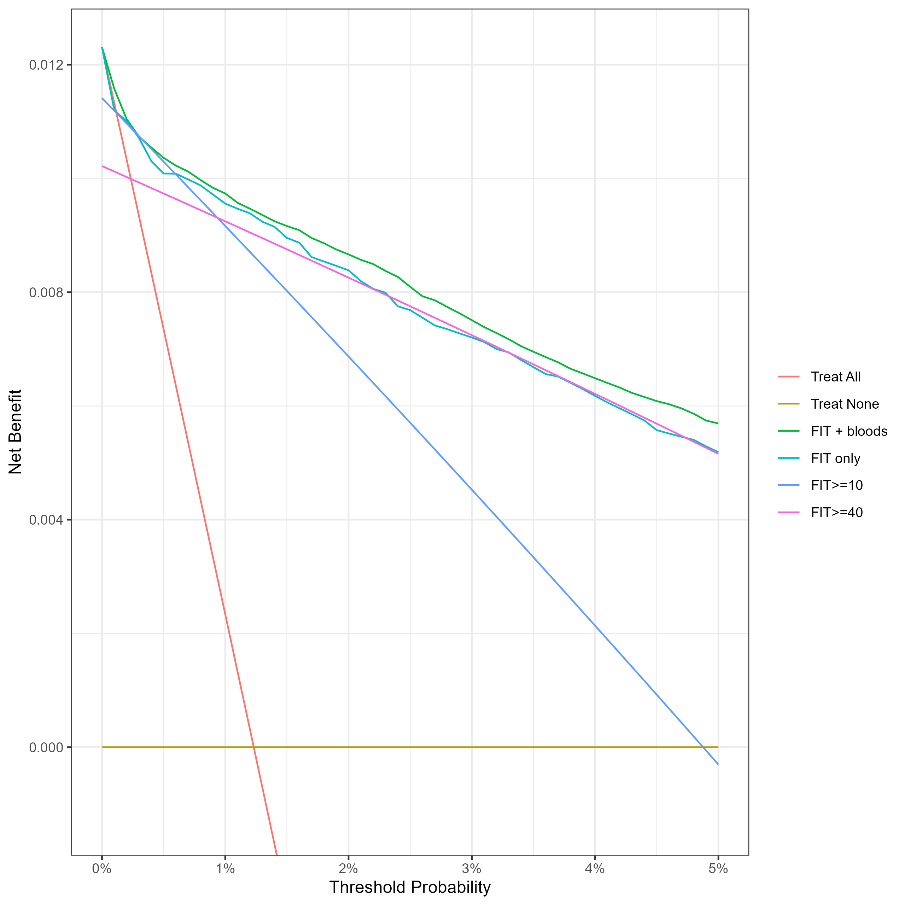

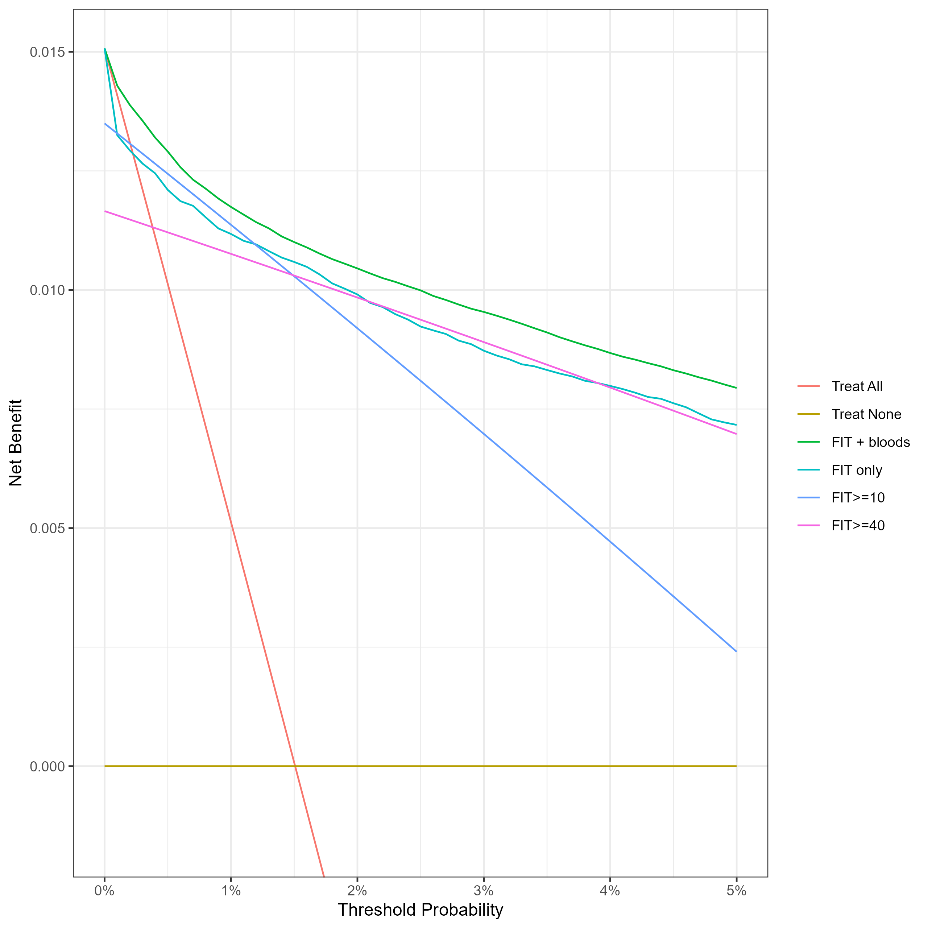


###### Table S10 Extrapolating true and false positive and negative rates from logistic model to 100,000 FIT tests in the validation cohort

|  | Derivation. FIT tests 1st November 2016- 30th November 2021 | | | | Validation: FIT tests 1st December 2021 - June 1st 2022 | | | |
| --- | --- | --- | --- | --- | --- | --- | --- | --- |
| Cancer risk threshold | **Colonoscopies performed for patients above predicted threshold** | **Detected cancer cases**  **(True positives)** | **Missed cancer cases**  **(False negatives)** | **Negative colonoscopies**  **(False positives)** | **Colonoscopies performed for patients above predicted threshold** | **Detected cancer cases**  **(True positives)** | **Missed cancer cases**  **(False negatives)** | **Negative colonoscopies**  **(False positives)** |
| Developed model with blood tests, FIT, age and sex | | |  |  |  |  |  |  |
| 0% (refer everyone) | 100000 | 1507 (1379 to 1633) | 0 (0 to 0) | 98493 (98367 to 98621) | 100000 | 1230 (1180 to 1280) | 0 | 98800 (98700 to 98800) |
| 0.6% | 21314 (20847 to 21764) | 1378 (1256 to 1502) | 129 (93 to 169) | 19936 (19485 to 20379) | 17600 (17400 to 17800) | 1120 (1070 to 1170) | 108 (92 to 125) | 16500 (16300 to 16700) |
| 1% | 16724 (16310 to 17142) | 1330 (1209 to 1452) | 178 (134 to 225) | 15395 (14992 to 15799) | 14100 (13900 to 14300) | 1100 (1060 to 1160) | 126 (109 to 143) | 13000 (12800 to 13100) |
| 2% | 12105 (11744 to 12457) | 1266 (1151 to 1385) | 241 (190 to 295) | 10838 (10496 to 11174) | 10400 (10200 to 10500) | 1060 (1010 to 1110) | 174 (154 to 194) | 9340 (9200 to 9470) |
| 3% | 9716 (9386 to 10055) | 1217 (1101 to 1332) | 291 (234 to 351) | 8499 (8194 to 8814) | 8330 (8190 to 8460) | 978 (929 to 1030) | 252 (230 to 275) | 7350 (7230 to 7480) |
| FIT, age and sex | | |  |  |  |  |  |  |
| 0% (refer everyone) | 100000 | 1507 (1379 to 1633) | 0 (0 to 0) | 98493 (98367 to 98621) | 100000 | 1230 (1180 to 1280) | 0 | 98800 (98700 to 98800) |
| 0.6% | 21682 (21250 to 22117) | 1370 (1247 to 1493) | 137 (99 to 178) | 20312 (19897 to 20739) | 20500 (20400 to 20700) | 1140 (1090 to 1190) | 95.7 (80.7 to 111) | 19400 (19200 to 19600) |
| 1% | 17297 (16900 to 17694) | 1318 (1198 to 1437) | 190 (146 to 237) | 15980 (15594 to 16371) | 16000 (15900 to 16200) | 1110 (1060 to 1160) | 120 (103 to 137) | 14900 (14800 to 15100) |
| 2% | 12492 (12135 to 12839) | 1242 (1125 to 1358) | 266 (213 to 321) | 11250 (10914 to 11586) | 12100 (11900 to 12200) | 1060 (1010 to 1110) | 168 (148 to 186) | 11000 (10800 to 11100) |
| 3% | 10070 (9754 to 10391) | 1198 (1084 to 1312) | 310 (251 to 371) | 8872 (8571 to 9179) | 10000 (9910 to 10200) | 1020 (970 to 1060) | 215 (194 to 237) | 9030 (8900 to 9180) |
| FIT only model |  |  |  |  |  |  |  |  |
| FIT ≥ 10 | 22427 (21989 to 22865) | 1350 (1230 to 1469) | 158 (117 to 202) | 21077 (20654 to 21504) | 30500 (30300 to 30700) | 1140 (1090 to 1190) | 89.6 (75.9 to 105) | 29300 (29100 to 29500) |
| FIT ≥ 40 | 10061 (9740 to 10377) | 1166 (1055 to 1280) | 342 (280 to 403) | 8895 (8597 to 9199) | 10700 (10500 to 10800) | 1020 (974 to 1070) | 209 (187 to 231) | 9630 (9490 to 9760) |
